# Special Issue: Rheology and Quality Research of Cereal-Based Food

**DOI:** 10.3390/foods9111517

**Published:** 2020-10-22

**Authors:** Anabela Raymundo, María Dolores Torres, Isabel Sousa

**Affiliations:** 1 LEAF—Linking Landscape, Environment, Agriculture and Food, Research Center of Instituto Superior de Agronomia, Universidade de Lisboa, Tapada da Ajuda, 1349-017 Lisboa, Portugal; isabelsousa@isa.ulisboa.pt; 2 Department of Chemical Engineering, Science Faculty, Universidade de Vigo (Campus Ourense), As Lagoas, 32004 Ourense, Spain; matorres@uvigo.es

**Keywords:** gluten-free products, dynamic oscillatory shear measurements, pasting profile, rheology, texture, X-ray diffraction, red kidney bean, microalga *Tetraselmis chuii*, acorn flour, tamarind gum, yogurt, tomato seed flour, antioxidants, phenolics, fibre-rich ingredient, ball milling

## Abstract

New trends in the cereal industry deal with a permanent need to develop new food products that are adjusted to consumer demands and, in the near future, the scarcity of food resources. Sustainable food products as health and wellness promoters can be developed redesigning traditional staple foods, using environmentally friendly ingredients (such as microalgae biomass or pulses) or by-products (e.g., tomato seeds) in accordance with the bioeconomy principles. These are topics that act as driving forces for innovation and will be discussed in the present special issue. Rheology always was the reference discipline to determine dough and bread properties. A routine analysis of cereal grains includes empirical rheology techniques that imply the use of well-known equipment in cereal industries (e.g., alveograph, mixograph, extensograph). Their parameters determine the blending of the grains and are crucial on the technical sheets that determine the use of flours. In addition, the structure of gluten-free cereal-based foods has proven to be a determinant for the appeal and strongly impacts consumers’ acceptance. Fundamental rheology has a relevant contribution to help overcome the technological challenges of working with gluten-free flours. These aspects will also be pointed out in order to provide a prospective view of the relevant developments to take place in the area of cereal technology.

## 1. Introduction

Innovation plays a key role in the current development of food companies. Food trends are launched every year, allowing stakeholders to be aligned around common axes on consumer’s preferences.

The most recent trends in the food industry [1] are focused on foods of vegetable origin, and commitment to sustainability stands out. The increase in the trend of personalized diet (“good for me”) is also noteworthy, which includes gluten-free products or foods with a direct impact on health and well-being (rich in bioactive compounds).

It is well known that products with the same chemical composition can present very different structures that are built up by processing techniques, resulting in differently perceived texture and sensory properties. Rheology has been the reference discipline for the food cereal industries since the very start of quality control. Food macromolecules (proteins and polysaccharides) are the major players for the creation of relevant food structures, such as dough and crisp snacks. The development of gluten-free products using alternative proteins and polysaccharides, nutritious mixtures of cereals and pulses to replace meat protein, as well as the use of food industry by-products, such as tomato seeds as a source of these structuring biopolymers (pectins), are some other challenges in creating innovative food products. Sustainability in the production of the food ingredients and the economic viability of their production and subsequent transformation into fair-traded, well-accepted food products, are essential for the progress of the cereal industry, with a relevant impact on human wellness and progress. The use of poorly exploited food sources, such as microroalgae, in cereal products is also an opportunity to explore. This approach includes the design of added value food products and relevant nutritional benefits. The use of ancient flours that have fallen out of use, such as acorn flour or ancient wheat varieties with low-gluten proteins, could be a sustainable strategy to enhance cereal products with an important source of bioactivities and fiber.

Finally, consumer attitude toward new food products is a relevant issue for the success of innovations and should be considered for food products that are close to the market. In this sense, the sensory evaluation of innovative products, in the preliminary stage of the development process, is an important tool in order to predict their final acceptance of the market.

## 2. Contributions

Gluten-free foods stand out in terms of food trends in the field of cereal technology. These types of products are on the agenda of the most important food companies and many research groups. This results from the steady increase of the gluten-free products market due to the growing number of individuals diagnosed with some type of gluten sensitivity. About 38% of consumers are avoiding or limiting gluten-free foods [2].

Generally, gluten-free foods are nutritionally unbalanced in terms of lipids, fibers, minerals, and vitamins. This can be critical for the celiac patients, who have co-pathologies and therefore need a nutritionally balanced diet [3].

As bakery products are traditionally produced with gluten flours, they have undergone extensive redesign in order to optimize their production from gluten-free flours. In this context, bread takes on a considerable prominence. Several works on this theme have been published in recent years [4,5,6], which are related to the incorporation of underexplored ingredients and by-products of the food industry, in order to improve the rheological and texture properties, nutritional performance, and sensory appreciation. In the present special issue, relevant studies related to gluten-free products are present.

Nunes et al. (2020) [7] evaluated the impact of *Tetraselmis chuii* microalgae incorporation on the structure, color, and bioactivity of a gluten-free (GF) bread formulation based on rice, buckwheat flour, and potato starch to increase nutrition, using hydroxipropylmethylcellulose (HPMC) as a structuring agent. The dough pasting profile assessed by Microdough-Lab and shear oscillatory measurements was conducted to evaluate the dough structure. Physical properties of the loaves, total phenolic content (*Folin–Ciocalteu*), and antioxidant capacity (DPPH and FRAP methods) of the bread extracts were assessed. Promising results related with the use of *T. chuii* as a sustainable ingredient in GF bread formulation were found, with a positive impact on the structure and antioxidant capacity and an innovative green appearance. This microalgae presented different behavior, according to the incorporation level: below 2%, *T. chuii* proteins destabilize the structure developed by starch and HPMC, and smaller bread volume was obtained, which was associated with a more compact crumb and harder properties. However, for higher levels of incorporation (4%), the microalgal proteins with starch and HPMC build up another type of structure, which is characterized by higher values of the viscoelastic functions (G‘ and G”) producing higher bread volume and a softening effect. There was evidence that the structure of 4% *T. chuii* bread is competitive with the control bread (with no biomass addition), in terms of structure and with a boost in nutritional performance, despite having revealed a weak acceptance by a non-targeted sensory panel.

Martins et al. (2020) [8] studied the possibility of using acorn flour, an under-exploited GF raw material, in a similar formulation as the one developed by Nunes et al. (2020). This flour was tested in order to improve dough rheology, following also market trends of sustainability and fiber-rich ingredients. Acorn flour significantly affected the rheology properties of the doughs. An impact on the dough’s mixing and pasting curves, an improvement of the texture parameters (firmness and cohesiveness) and the viscoelasticity of the fermented dough were highlighted. In this way, the role of dough characterization by rheology tools was evidenced, as being determinant for the optimization of new food products. According to small amplitude oscillatory shear measurements, all the GF doughs studied exhibit a weak gel-elastic-like behavior with G’ values higher than G” and frequency dependent. Acorn flour incorporation caused the acidification and increased the darkness of the dough, which can have a positive impact in terms of sensory appreciation of the bread. Therefore, it was stated that acorn flour can be a very promising ingredient to improve both the rheological GF dough properties and nutritional GF bread quality, in particular dietary fiber content, which is a really important nutrient in special requirement diets. 

In line with the work already presented on the nutritional enrichment of GF bread, Graça et al. (2020) [9] studied the possibility of enriching a similar type of bread with yogurt. Following this strategy, the low functional and nutritional properties of GF bread can be minimized, using dairy protein. Fresh yogurt represents an interesting ingredient since in addition to being an important protein source, it also provides polysaccharides and minerals that have the potential to mimic the gluten network, while improving the nutritional value of gluten-free products. Gluten-free bread formulations, with different levels of yogurt addition (5% up to 20%, *weight*/*weight*), were evaluated, using dough rheology measurements and baking quality parameters. It was shown that the functionality of gluten-free breads, in terms of bread-making performances, quality parameters, and nutritional profile can be successfully improved by the addition of fresh yogurt, resulting in a significant improvement in the overall quality of the GF yogurt-breads. Linear correlations between bread firmness, specific volume with flow behavior, and viscoelastic functions were found, supporting the results obtained. These correlation can assume a considerable importance in terms of the bakery industry and for future studies in this area. Yogurt was shown to be a potential ingredient to improve the quality of gluten-free breads, resulting in softer breads with higher volume and lower staling rate, compared to control bread. In relation to the nutritional composition, yogurt addition was revealed to be an attractive ingredient to enhance the nutritional value of GF breads: an increase in the protein and mineral contents and a reduction in carbohydrates was found, with a good chance to improve the daily diet of celiac people. 

Hong and Kweon (2020) [10] presented another study on gluten-free rice bread, incorporating tamarind gum. In this work, the importance of optimizing the formulation and processing conditions is highlighted when combining new ingredients for product design. An experimental factorial design was used and revealed to be useful for the optimization process, minimizing the number of experiments and emphasizing the weight of each independent variable in the explanation of the process. Gum concentration (GC), water amount (WA), mixing time (MT), and fermentation time (FT) were selected as factors, and two levels were used for each factor. WA and FT were identified as the most significant factors to determine the quality of GF rice bread with tamarind gum. Therefore, proper control over the water content and fermentation time can maximize the bread volume and minimize the firmness of the bread. The addition of an anti-staling enzyme was also studied and proved to be effective in retarding the retrogradation enthalpy and decrease of bread firmness. Using an optimized formula and processing factors for gluten-free rice bread with the combined addition of tamarind gum and an anti-staling enzyme (maltogenic amylase) can be applied successfully in commercially manufactured gluten-free rice bread. 

In addition to gluten-free bread, several formulations of bakery products, based on GF flours, have appeared on the market. Chompoorat et al. (2020) [11] studied the impact of rice flour addition on red kidney bean (RKB) cupcakes. The incorporation of rice flour promoted an increase in the degree of structuring of the cupcake dough and an improvement in the texture properties of the RKB cupcakes. It is important to note that also for this type of matrix, the use of fundamental rheological techniques such as dough linear viscoelastic behavior and empirical tests such as texture characterization were crucial to optimizing the final product. Rice flour addition in gluten-free RKB flour increased the batter’s solid-like and viscous behavior, batter consistency, inflection of gelatinization and temperature, and produced a softer cupcake texture. The activation energy of gelatinization also increased with rice incorporation, as well as the cupcakes’ macrostructural characteristics. The potential of RKB as a functional ingredient and its improvement in cupcake application with the addition of rice flour was highlighted. 

Arribas et al. (2019) [12] studied another relevant GF product: an extruded rice snack. This type of product, in addition to being part of the current trend in the consumption of GF cereal foods, assumes an important position in the snacking food trend [13]. Snacking has intensively grown in the last few years and is associated with the growth of new forms of consumption associated with more dynamic lifestyles. The authors evaluated the impact of adding two legumes, bean and carob fruit flours, on the physical properties and bioactivity of GF puffed snacks. The fortification with carob fruit flour improved their textural attributes and did not significantly affect their overall quality. The extrusion had positive implications in terms of the nutrition and availability of bioactive compounds, as well as good acceptance in sensory terms. All the experimental extrudates had higher amounts of bioactive compounds than the commercial extruded rice. This process affected phytochemicals to a different extent. While total α-galactosides and phenols increased, the phytic acid was reduced, and the lectins and protease inhibitors were eliminated. The content of bioactive compounds present in these extrudates might be enough to promote health-associated functions. Moreover, the absence of lectins and protease inhibitors enhanced the nutritional quality of the extrudates. The developed snacks would be of interest to both health-conscious consumers and the food industry.

The development of gluten-free bakery products is a major challenge, which can be overcome through several strategies that were already presented in the works mentioned above. The use of hydrocolloids that are capable of creating a structure that mimics the gluten matrix is often followed. Nuvoli et al. (2020) [14] studied the effect of ball milling treatment on different types of hydrocolloids in a corn starch–rice flour system. Guar gum (GG), tara gum (TG), and methylcellulose (MC) were the hydrocolloids studied, and they were previously analyzed to assess their potential interactions with starch components, when they are used alone or in blends in a corn starch–rice flour system. Based on X-ray diffraction (XRD) experiments and gelling rheology characterization, it could be stated that the ball milling treatment affected the structure of the tested hydrocolloids and, in turn, the viscosity of their aqueous solutions in different ways. In fact, ball milling caused a reduction in the crystallin domain and, in turn, a diminished viscosity of the GG aqueous solutions. Despite an increase in its flow properties (viscosity), effects on TG were minimal, while the milled MC exhibited reduced crystallinity but similar viscosity. When both milled and un-milled hydrocolloids were individually added to the starch–flour system, the pasting properties of the resulting mixtures seemed to be affected by the type of hydrocolloid added, rather than by the structural changes induced by the treatment. All hydrocolloids increased the peak viscosity of the binary blends (especially pure GG). However, only milled and un-milled MC showed values of setback and final viscosity similar to those of the individual starch. Ball milling seemed to be more effective when two combined hydrocolloids (milled GG and MC) were simultaneously used. No significant differences were observed in the viscoelastic properties of the blends, except for un-milled GG/starch, milled TG/starch, and milled MC/milled TG/starch gels. The work presented was considered by the authors as an initial study, recognizing the need to deepen the theme. In this way, in future studies, the relevance of using the ball milling treatment, in the development of gluten-free bakery products, using hydrocolloids–starch systems will be clarified.

The enrichment of bakery goods, with and without gluten, by means of the incorporation of by-products from the food industry, has assumed special relevance, in recent years, taking into account the principles of the circular economy. Mironeasa and Codina (2019) [15] studied the utilization of a very relevant by-product of the food industry: tomato seed flour (TSF) produced. This work also highlighted the importance in the rheological and microstructure characterization of the dough on the bakery products development process. Rheology methods through the Mixolab device, dynamic rheology, and epifluorescence light microscopy (EFLM) were used to characterize the dough obtained from different formulations. From the Mixolab results, it was noticed that replacing wheat flour with TSF increased the dough development time, stability, and viscosity during the initial heating–cooling cycle and decreased alpha amylase activity. The dynamic rheological data showed that the viscoelastic moduli (G’ and G”) increased with the level of TSF addition. Creep recovery tests of the samples indicated that the dough elastic recovery was at a high percentage after stress removal for all the samples in which TSF was incorporated in wheat flour. Using EFLM, all the samples seemed homogeneous showing a compact dough matrix structure. The parameters measured with Mixolab during mixing were in agreement with the dynamic rheological data and in accordance with the EFLM structure images. The authors show a correlation between the mixing properties and the viscoelastic behavior for the studied system (with gluten), as was also verified by Graça et al. (2020) [6], of GF bread. 

The results presented in the different works of this special issue are useful for bakery producers to develop new products with the highest nutritional value, respecting the major key trend of sustainability and aligning with the major food trend to answer the consumer’s actual needs.

## References

[1] EUFIC Food Choice—Why Do We Eat What We Eat. https://www.eufic.org/en/food-safety/healthy-living/category/food-choice.

[2] Hendry N. (2019). Innovation Opportunities across the Global Food Landscape.

[3] Missbach B., Schwingshackl L., Billmann A., Mystek A., Hickelsberger M., Bauer G., König J. (2015). Gluten-free food database: The nutritional quality and cost of packaged gluten-free foods. PeerJ.

[4] Wang K., Lu F., Li Z., Zhad L., Han C. (2017). Recent developments in gluten-free bread baking approaches: A review. Food Sci. Technol. Campinas..

[5] Beltraão-Martins R., Gouvinhas I., Nunes M.C., Peres J., Raymundo A., Barros A. (2020). Acorn Flour as a Source of Bioactive Compounds in Gluten-Free Bread. Molecules.

[6] Graça C., Fradinho P., Sousa I., Raymundo A. (2018). Impact of *Chlorella vulgaris* on the rheology of wheat flour dough and bread texture. LWT—Food Sci. Technol..

[7] Nunes M.C., Fernandes I., Vasco I., Sousa I., Raymundo A. (2020). *Tetraselmis chuii* as a Sustainable and Healthy Ingredient to Produce Gluten-Free Bread: Impact on Structure, Colour and Bioactivity. Foods..

[8] Beltrão-Martins R., Nunes M.C., Ferreira L.M., Peres J.R.N.A., Barros A.I., Raymundo A. (2020). Impact of Acorn Flour on Gluten-Free Dough Rheology Properties. Foods.

[9] Graça C., Raymundo A., Sousa I. (2020). Yogurt as an Alternative Ingredient to Improve the Functional and Nutritional Properties of Gluten-Free Breads. Foods.

[10] Hong Y.-E., Kweon M. (2020). Optimization of the Formula and Processing Factors for Gluten-Free Rice Bread with Tamarind Gum. Foods.

[11] Chompoorat P., Kantanet N., Hernández Estrada Z.J., Rayas-Duarte P. (2020). Physical and Dynamic Oscillatory Shear Properties of Gluten-Free Red Kidney Bean Batter and Cupcakes Affected by Rice Flour Addition. Foods.

[12] Arribas C., Cabellos B., Cuadrado C., Guillamón E., Pedrosa M. (2019). Bioactive Compounds, Antioxidant Activity, and Sensory Analysis of Rice-Based Extruded Snacks-Like Fortified with Bean and Carob Fruit Flours. Foods.

[13] Mordor Intelligence Free-From Food Market—Growth, Trends, and Forecast (2020–2025). https://www.mordorintelligence.com/industry-reports/free-from-food-market.

[14] Nuvoli L., Conte P., Garroni S., Farina V., Piga A., Fadda C. (2020). Study of the Effects Induced by Ball Milling Treatment on Different Types of Hydrocolloids in a Corn Starch–Rice Flour System. Foods.

[15] Mironeasa S., Codină G.G. (2019). Dough Rheological Behavior and Microstructure Characterization of Composite Dough with Wheat and Tomato Seed Flours. Foods.

